# A Root-Colonizing Pseudomonad Lessens Stress Responses in Wheat Imposed by CuO Nanoparticles

**DOI:** 10.1371/journal.pone.0164635

**Published:** 2016-10-24

**Authors:** Melanie Wright, Joshua Adams, Kwang Yang, Paul McManus, Astrid Jacobson, Aniket Gade, Joan McLean, David Britt, Anne Anderson

**Affiliations:** 1 Department of Biological Engineering, Utah State University, Logan, Utah, 84322 4105, United States of America; 2 Department of Biology, Utah State University, Logan, Utah, 84322 5305, United States of America; 3 Utah Water Research Laboratory, Utah State University, Logan, Utah, 84321, United States of America; 4 Plants Soils and Climate, Utah State University, Logan, Utah, 84322 4820, United States of America; Estacion Experimental del Zaidin, SPAIN

## Abstract

Nanoparticle (NPs) containing essential metals are being considered in formulations of fertilizers to boost plant nutrition in soils with low metal bioavailability. This paper addresses whether colonization of wheat roots by the bacterium, *Pseudomonas chlororaphis* O6 (*Pc*O6), protected roots from the reduced elongation caused by CuO NPs. There was a trend for slightly elongated roots when seedlings with roots colonized by *Pc*O6 were grown with CuO NPs; the density of bacterial cells on the root surface was not altered by the NPs. Accumulations of reactive oxygen species in the plant root cells caused by CuO NPs were little affected by root colonization. However, bacterial colonization did reduce the extent of expression of an array of genes associated with plant responses to stress induced by root exposure to CuO NPs. *Pc*O6 colonization also reduced the levels of two important chelators of Cu ions, citric and malic acids, in the rhizosphere solution; presumably because these acids were used as nutrients for bacterial growth. There was a trend for lower levels of soluble Cu in the rhizosphere solution and reduced Cu loads in the true leaves with *Pc*O6 colonization. These studies indicate that root colonization by bacterial cells modulates plant responses to contact with CuO NPs.

## Introduction

Plants exist in association with microbes, which include root-colonizing bacteria that improve the resilience of plants to stress conditions [1]. Use of such beneficial microbes could result in higher sustainable crop production on marginal soils [2] including those that are contaminated by metals [3–5]. Increases in metal uptake into hyperaccumulating plants during phytoremediation involve their association with certain beneficial microbes [5]. Interestingly, many of the bacteria that improve phytoremediation have common traits: the production of the plant hormone, indoleacetic acid (IAA), and the secretion of the enzyme 1-aminocyclopropane-1-carboxylate (ACC) deaminase, and iron-chelating siderophores [5]. Degradation of the ethylene-precursor ACC, by the deaminase, is proposed to lessen stress associated with ethylene production induced in the plant by the metal challenge [6]. Chelation of metals by the secreted siderophores may affect bioavailability of the metal to the plant [7].

Studies of metal interactions with plants include metal and metal oxide nanoparticles (NPs); such NPs are being incorporated into a growing number of commercial products that will lead to their accumulation in the environment [8]. CuO NPs have diverse commercial applications, including potential use in agriculture as fertilizers or pesticides [9]. Inadvertent NP contamination may occur from their presence in field—applied biosolids [10]. CuO NPs cause dose-dependent toxicity in plants, commonly displayed as impaired root elongation, which is accompanied by increased plant Cu especially in the roots [11–16]. Uptake of CuO NPs and Cu released by dissolution of the NPs is supported by studies with several different plants [11, 12, 14].

This paper investigates whether a beneficial root-colonizing microbe, *Pseudomonas chlororaphis* O6 (*Pc*O6), modulates the responses of wheat challenged by CuO NPs. The isolate, from roots of dry land commercial wheat [17]. boosts plant resistance to pathogens and to abiotic stress [18]. *Pc*O6 has two traits in common with the microbes that influence plant-metal responses: IAA and siderophore production [19, 20]. The isolate does not possess the deaminase according to the annotation of its genome [21]. CuO NPs cause dose-dependent toxicity in planktonic cells; sub-lethal levels change the flux of metabolites such that IAA production increases but siderophore secretion decreases [19].

The studies in this paper were designed to resolve the outcomes of CuO NP-challenge to plant roots when colonized with *Pc*O6. We determined whether *Pc*O6 modified the inhibition of root elongation for wheat seedlings grown in sand with a sublethal dose of CuO NPs and whether root colonization by the bacterium was reduced by the NPs. Physical attachment of *Pc*O6 cells on root surfaces was confirmed by SEM microscopy. NP-induced phytotoxicity may include responses to reactive oxygen species (ROS) induced in cells by CuO NPs [22, 23]. However, *Pc*O6 cells possess an array of enzymes such as catalases, peroxidases and superoxide dismutases [24] that may help protect against the mobile ROS, hydrogen peroxide, generated in plant root cells. Consequently seeds were inoculated with a *gacS* mutant of *Pc*O6 that colonizes plant roots effectively but which has enhanced sensitivity to ROS stress; several isozymes of peroxidase and superoxide dismutase are absent in the *gacS* mutant [24, 25]. Additionally fluorescent probes were employed to detect whether roots colonized by *Pc*O6 had altered ROS production when grown with exposure to CuO NPs. CellROX Deep Red was used to examine the intracellular accumulation of ROS [26], fluorescence from C11-BODIPY^581-591^ was imaged to determine the production of lipid peroxides [27] and the accumulation of superoxide anion was studied with fluorescence from dihydroethidium (DHE) [28]. Another dye, SYTOX Blue, was used to determine how treatments modified cell death at the surface of the root tips [29].

Changes in the release of organic acids in the plant rhizosphere occur as part of the mechanisms associated with tolerance to toxic metal challenge [30]. When challenged with Cu and Al ions, wheat roots differentially release malate and citrate [31]. However, these plant root exudates are used as carbon sources for growth by root-associated microbes [32, 33], as is demonstrated for wheat root exudates and *Pc*O6 [34]. Consequently, we determined whether the composition of organic acids detected in the root rhizosphere was changed due to *Pc*O6-root colonization with and without exposure to CuO NPs. The effects of *Pc*O6 colonization on the levels of soluble Cu in the rhizosphere solution and Cu loading into the shoots were measured.

To understand the impact of the interactions on root cell function, we examined the levels of gene expression upon exposure to *Pc*O6 or CuO NPs alone and in combination. The plant genes selected for study included those that function in general stress, detoxification and metal- associated functions as well as transcriptionally-active factors. Such genes are found to be activated in plant responses to ZnO NPs [35].

## Materials and Methods

### Microbial culture and plant growth

Cultures of *Pc*O6 and its *gacS* mutant [36] were maintained at -80°C in 15% sterile glycerol. Cells were grown on a minimal medium [37] for 14 h prior to use as the seed inoculum.

Wheat seedlings, a hard red winter wheat cultivar Doloris, were grown for 7 d in sterilized sand (300 g) with and without 10, 100 mg or 300 mg Cu from CuO NPs/kg sand and 50 mL sterilized water. The CuO nanoparticles, sourced from Sigma, were characterized for size and chemical composition as reported previously [12, 22, 38]. The wheat seed was surface sterilized in 10% hydrogen peroxide for 10 min prior to rinsing extensively with sterile water and planting into the sterilized growth matrix. Surface—sterilized seeds were inoculated by immersion in suspensions of *Pc*O6 or its *gacS* mutant (10^6^ colony forming units/ml) for 10 min before being shaken dry and transfer to the growth boxes. Each box was planted with 20 seeds. To examine the extent of colonization of wheat seedling roots, the roots at harvest were transferred to 10 mL sterile water and serial dilutions plated onto rich Luria-Bertani medium plates. Growth of *Pc*O6 wild type strain on this medium was as orange colonies whereas the colonies of the *gacS* mutant had no coloration [37]. Root and shoot lengths were measured for each of the seedlings that were grown 5 /box with 5 replicates of each treatment. Each study was repeated at least three independent times.

### Microscopic detection of ROS

For the microscopic studies, the seedlings were grown in 300 g of sand wetted with 50 mL water with and without addition of CuO NPs and with and without colonization with *Pc*O6. Root tips were treated with fluorescent detector dyes immediately after harvest at 7 d. Images were generated from at least three roots/ study and each of the treatments were replicated in three separate studies.

Production of intracellular ROS was detected using CellROX Deep Red (Life Technologies Carlsbad CA) [26]. The manufacturers indicate this dye is oxidized preferentially by hydroxyl radicals and superoxide anions rather than hydrogen peroxide or activated nitrogen species. The stock solution was diluted 5 fold and 3 μL transferred to 1.5 mL sterile distilled water to generate the working concentration, 1 μM. The root tips were directly transferred to the dye solution for 30 min before being imaged by confocal microscopy using a LSM710 (Zeiss Microscopy) with excitation 633 nm and emission between 638 and 738 nm. Raw images were analyzed for dye intensity using a 100 pixel square in ImageJ. Images used in the figures have brightness and contrast enhancements.

To assess accumulation of superoxide anion, as suggested by studies with metal ions in Arabidopsis [39] the dye, dihydroethidium (DHE) (Life Technologies, Carlsbad CA), was used [28]. The oxidized ethidium product locates with the cellular DNA and is visualized as a red fluorescence. The root tips were exposed to a final concentration of 20 μM DHE obtained by dilution of the DMSO stock in sterile water. After 15 min exposure LSCM images were obtained without rinsing the tips using excitation at 514 nm and emission at 556–685 nm. Raw images were analyzed for dye intensity using a 100 pixel square in ImageJ. Images used in the figures have brightness and contrast enhancements.

The accumulation of lipid peroxides was detected with C-11 BODIPY^581/591^ (Life Technologies, Carlsbad CA) [26, 27]. Root tips were immersed in 10 μM dye prepared by dilution of the DMSO stock solution in sterile distilled water for 30 min and briefly rinsed with distilled water. LSCM images were obtained at Ex/Em: 488/493-551 nm and 561/591-687 nm. Images used in the figures have brightness and contrast enhancements.

To visualize surface root cells with defective plasma membranes, the dye, SYTOX Blue, (Life Technologies/Thermo Fisher Scientific Carlsbad CA) was used; this dye is reported only to stain cells with damaged plasma membranes permitting the dye to interact with DNA in the nuclei and to emit fluorescence when excited. The stock dye was diluted to 500 μM according to manufacturer’s instructions before adding to the washed root tips at a final concentration of 1 μM. After 15 minutes the tips were mounted between a glass slide and coverslip and fluorescence was observed with the LSM 710 with excitation at 458 nm and emission at 463–509 nm. Autofluorescence of the root tips was examined using tissues not treated with SYTOX Blue. For each study at least three root tips were examined for one treatment and at least three independent studies were performed before representative images were selected. The images were uniformly processed for brightness and contrast.

### RNA extraction, reverse transcription, and quantitative real-time polymerase chain reaction (qPCR)

Total RNA was extracted using the RNeasy Mini Kit (Qiagen) from roots that had been frozen in liquid nitrogen and ground to a powder immediately upon harvest. RNA concentrations were quantified using a NanoDrop 100 and sample quality was qualified from the 260 nm / 280 nm ratio. Total RNAs extracted from samples were used for the synthesis of first-strand cDNAs by reverse transcriptase after DNase treatment. Reverse transcription was performed for 1.5 h at 42°C in a final reaction volume of 20 μL containing 1.5 μg of the purified total RNA, 4 μL of 5X reaction buffer (Promega, Madison, WI, USA), 5 μL of dNTPs (each 2 mM), 2 μL of 10 μM oligo dT, 0.5 μL of RNase Inhibitor (40 U / μl; Promega), and 1uL of AMV reverse transcriptase (200 U / μL; Promega). PCR with SYBR Green detection was conducted in a Bio-Rad iQ5 real-time PCR detection system (Bio-Rad Laboratories) in 96-well plates (Thermo Scientific). Aliquots (2 μL) of cDNA (20 ng / reaction) were used for template for qPCR reactions with the Bio-Rad SYBR Green PCR Master Mix (Bio-Rad Laboratories) and with primers at 500 nmol/L final concentration. Primers for the genes are listed in S1 Table. Amplification conditions for the PCR reactions were 95°C for 3 min, followed by 40 cycles of 95°C for 15 sec and 55°C for 30 sec, and a final melting curve analysis step from 55°C to 95°C. Gene expression levels were determined using the Delta-Delta-CT method. After normalization to an *ADP-*ribosylation factor control, the relative levels of gene expression were calculated [40].

### Assessment of metal concentration and organic acids in rhizosphere solutions and plant tissue

At harvest growth boxes with the plants left intact were amended with 40 mL water (2 ml/seedling) for 30 min. The plants were carefully removed and the wetted sand filtered with FGA filters with vacuum on a Buchner funnel. The filtrate was collected and filtered through 0.2 micron filters to remove debris and bacterial cells. The filtrates were stored frozen until assayed for organic acids using ion column chromatography with gradient elution with KOH and detection by electrical conductivity. A Dionex ICS-3000 equipped with a Dionex INPAc AS11HC analytical column was used for the chromatography. Appropriate standards were used to determine peak elution and concentration. For metal analysis with inductively coupled plasma mass spectrometry (ICP-MS) the samples were freed of NPs by their precipitation by centrifugation twice at 12,000 g. Coleoptiles were excised from the harvested shoot tissues manually and dried for analysis of metal without washing to remove surface accumulated materials. The true leaves which had been stripped of their coleoptiles were washed in sterile distilled water three times by immersion before drying and determination of metal levels by ICP-MS.

## Results

### Interaction of CuO NPs and *Pc*O6 colonization on wheat seedling growth and root surface colonization

**Fig 1:**
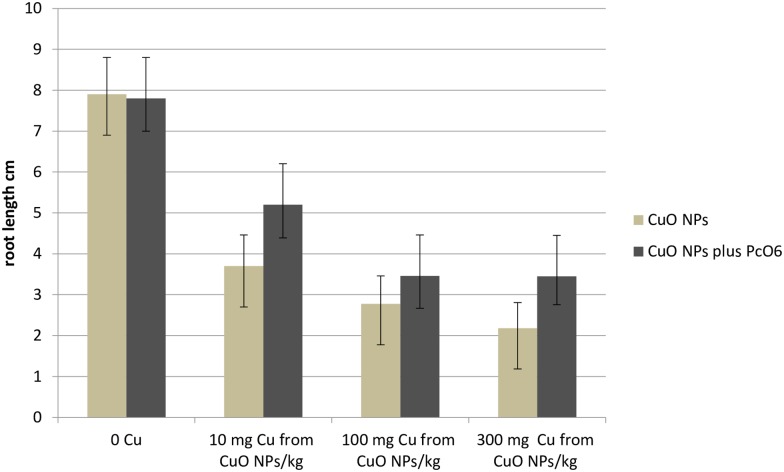
Effect of CuO NPs on wheat seedling root growth. Plants were grown for 7 d from seeds with and without inoculation by *Pc*O6 in sand amended with CuO NPs at the shown levels. Data are means with standard deviations from two independent studies each with 5 replicated growth boxes each containing 20 wheat seedlings. There are no significant differences (p = 0.05) between root length from plants with and without *Pc*O6 for any growth condition.

Colonization of root by *Pc*O6 in the presence of different doses of CuO NPs caused a trend, without statistical significance at p = 0.05, for increased root length from growth with CuO NPs without root colonization (Fig 1). Root colonization with *Pc*O6 in the presence of 300 mg Cu from CuO NPs /kg sand did not affect shoot length (S1 Fig).

Assessment of the levels of *Pc*O6-colonization of roots showed that exposure to CuO NPs had no statistical effect on bacterial cell density on the root. Roots were colonized with 6.1 ± 1.8 x 10^8^ cells/g wet weight of root without CuO NP exposure compared with 4.2 ± 2.0 x 10^8^ cells/g wet weight root with 300 mg Cu from CuO NPs /kg sand. Studies were extended to examine whether colonization by a *gacS* mutant of *Pc*O6 was affected by CuO NPs. Planktonic cells of this mutant were more sensitive to CuO NPs than the wild type strain. A dose of 200 mg Cu from CuO NPs /L reduced the culturability in a suspension of wild type cells from 8.0 ± 0.1 x 10^8^ to 0.72 ± 0.2 x 10^8^ cells/mL in I h compared with complete loss of culturability of the *gacS* mutant (i.e., 8.1 ± 0.1 x 10^8^ to 0 cells/mL). However, culturable cells of the *gacS* mutant were recovered from root surfaces at levels that were not statistically different whether the plants were grown with or without 100 or 300 mg Cu/kg sand from CuO NPs (S2 Fig).

### *Pc*O6’s influence on morphology of CuO NP-challenged roots

**Fig 2:**
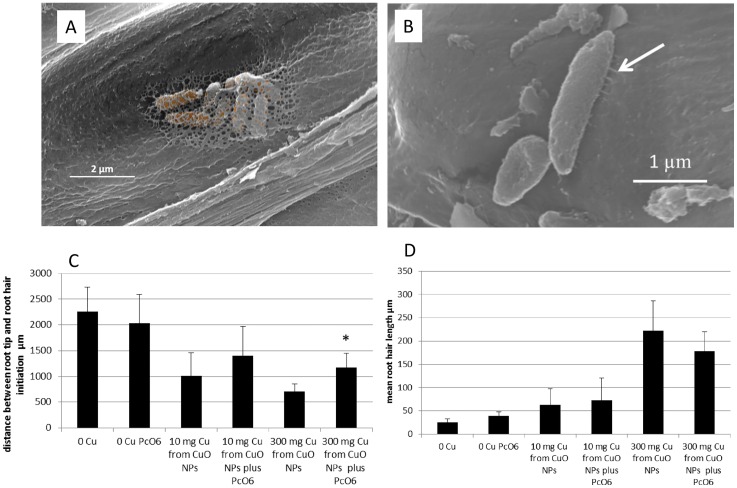
(A-D) Scanning electron microscope images of a wheat root surface grown in sand from a wheat seed inoculated with *Pc*O6 showing attached cells: (A) Cells are covered with extracellular materials and (B) Cells show small projections (see arrow) that could be pili, that attach to the root surface. Size bars are 2 μm for A and 1 μm for B. (C) Comparisons of the distance measured between root tips and the location of the first root hairs. Data are means with standard deviations of measurements from 10 different roots. The * indicates significant difference (p = 0.05) between the data from roots grown with and without *Pc*O6 colonization. (D) Comparisons of the length of the initial root hairs on roots grown with and without CuO NPs and colonization with *Pc*O6. Data are the means with standard deviations of measurement from 10 different roots. There was no significant difference between the control and *Pc*O6 colonized roots for any of the treatments.

Microscopy of the root tips confirmed the presence of *Pc*O6 cells attached to the root surface (Fig 2A and 2B). A cluster of cells embedded in matrix material on the root surface near the root tip is shown in Fig 2A. Other images showed less overlying matrix but short attachment structures, possibly pili, linking the bacterial cell to the root surface (Fig 2B). Microscopy of the root tips showed that the zone of differentiation into root hairs varied between 1,500 and 2,500 μm from the root tips for seedlings grown without CuO NPs, with and without colonization by *Pc*O6 (Fig 2C). However growth with CuO NPs reduced the distance between root tips and the start of epidermal cell differentiation into the root hairs starting as close as 500 μm. This distance was slightly elongated when the root were colonized with *Pc*O6 (Fig 2C). The engths of the first root hairs also were longer for the tips exposed to CuO NPs with or without *Pc*O6 colonization compared with lengths in control plants (Fig 2D).

### *Pc*O6 influence of uptake of Cu into shoots

**Fig 3:**
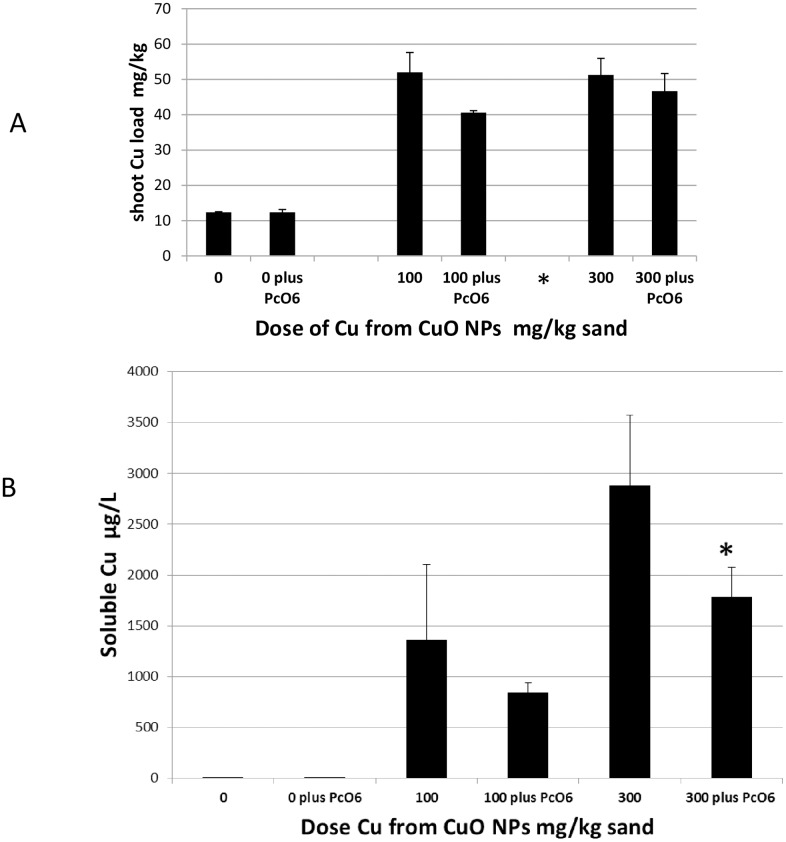
Effects of CuO NPs and *Pc*O6 colonization on loading of Cu into shoots (A) and soluble Cu in the rhizosphere solution (B). Data are means from two independent studies each with 5 replicate boxes containing 20, 7 d-old wheat seedlings. The * indicates significant difference (p = 0.05) between plants with and without *Pc*O6 colonization.

Root colonization with *Pc*O6 reduced the level of Cu measured in shoot tissues when plants were grown with CuO NPs (Fig 3A). Reduction was statistically significant with a dose of 100 mg/kg CuO NPs but not for the higher dose of 300 mg/kg CuO NPs. These data sets are for the true leaves of the wheat seedlings from which coleoptiles have been removed. The values for leaf accumulation are lower than those (>300 mg/kg) where total shoot tissue (i.e., true leaves plus the coleoptiles) was measured [12]. Surface loading of coleoptiles with Cu for plants grown with CuO NPs was demonstrated by measurement of this tissue without any water washes (S3 Fig). SEM imaging revealed deposits with the chemical signature of CuO on the outer surface of the unwashed coleoptile tissue. No such deposits were observed on the inner surfaces (S4 Fig). Coleoptile lengths were not reduced in plants grown with with CuO NPs, unlike the reduction observed in root elongation (S5 Fig). The inclusion of CuO NPs in the sand growth mixture increased the level of soluble Cu in the rhizosphere solution (Fig 3B). However, these levels were reduced with *Pc*O6 colonization of the roots, with significance for the 300 mg/kg dose.

### ROS formation in root tip cells

**Fig 4:**
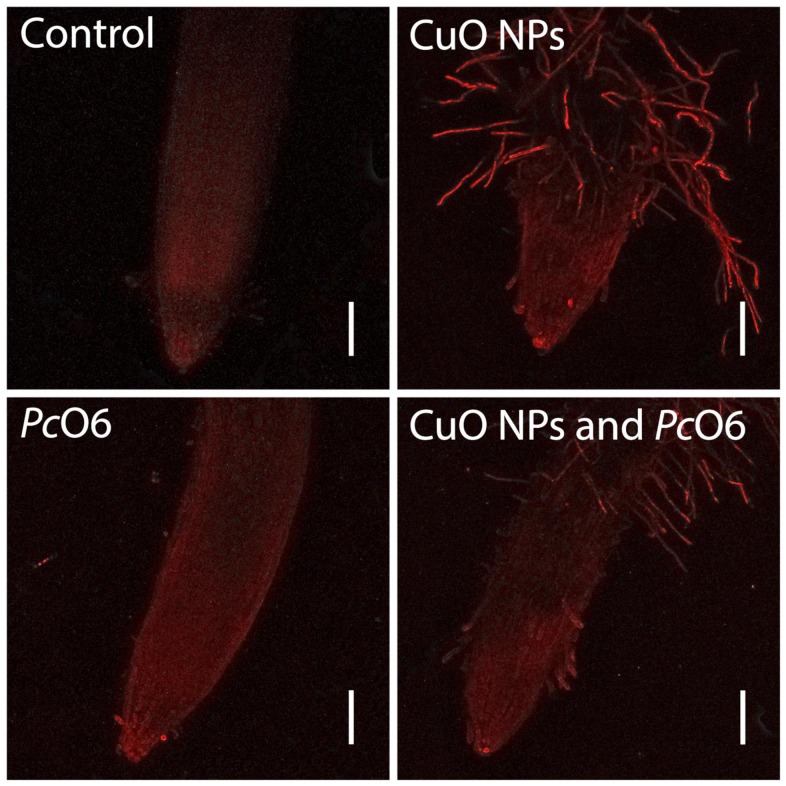
Confocal images examining reactive oxygen stress in wheat root tips of plants grown for 7 d with and without CuO NPs and root colonization with *Pc*O6: accumulation of intracellular ROS as determined by staining with CellROX Deep Red.

**Fig 5:**
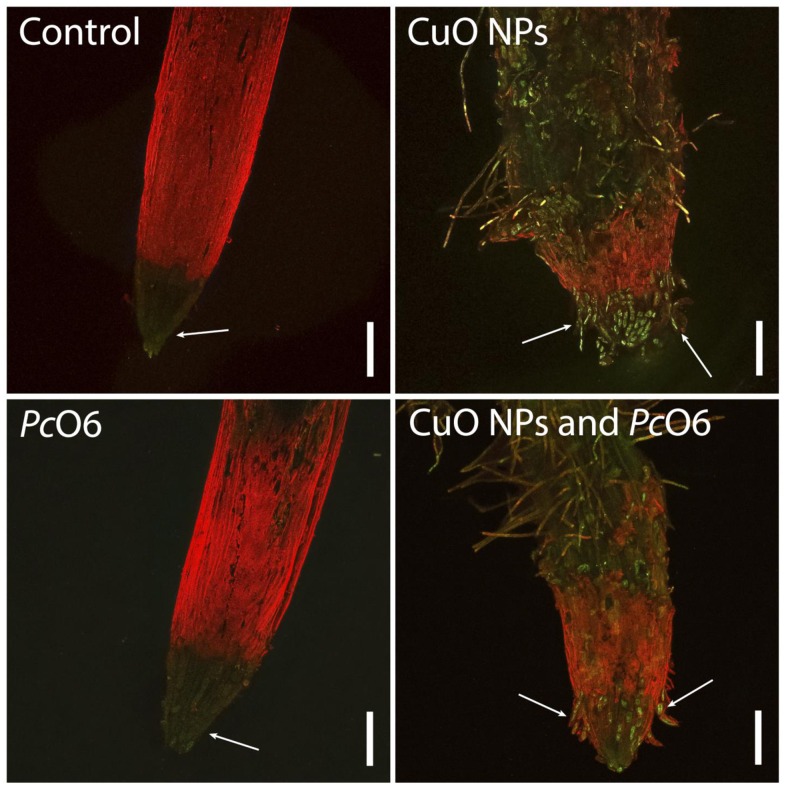
Confocal images examining reactive oxygen stress in wheat root tips of plants grown for 7 d with and without CuO NPs and root colonization with *Pc*O6: accumulation of lipid peroxides as determined by staining with C11-BODIPY.

**Fig 6:**
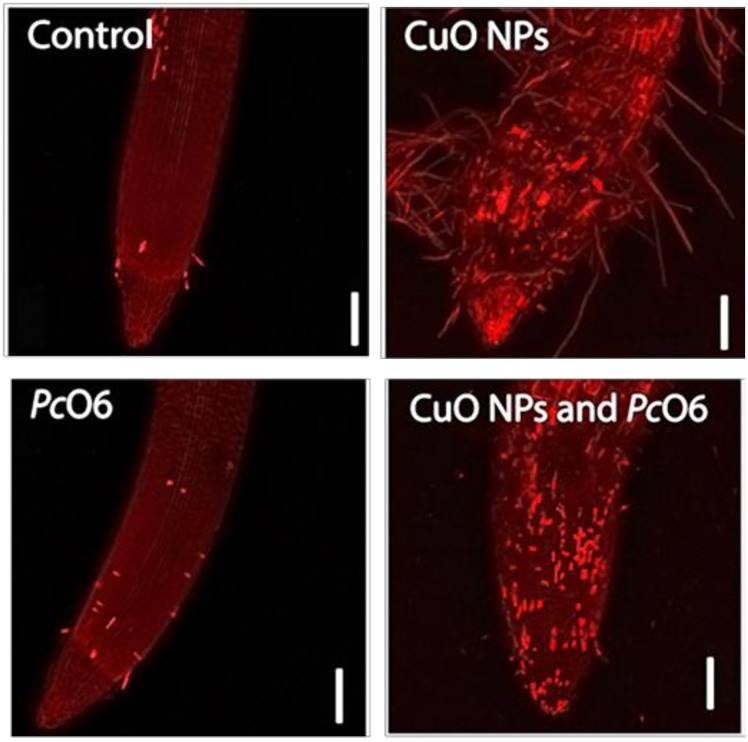
Confocal images examining reactive oxygen stress in wheat root tips of plants grown for 7 d with and without CuO NPs and root colonization with *Pc*O6: accumulation of superoxide revealed by treatments with DHE.

Changes in intracellular ROS, the presence of lipid peroxides and superoxide anions was examined using three dyes that changed in their fluorescence in response to ROS presence. There was no autofluorescence of the tissues under the imaging conditions for any of these indicators (S6 Fig). There was a slight increase in overall fluorescence for the root tips with *Pc*O6 colonization when treated with CellROX Deep Red (Fig 4) from the levels in noninoculated root tips. Increased red fluorescence indicative intracellular ROS accumulation was apparent in the root hairs for plants grown with CuO NPs and discrete cells in the root cap border cells with or without *Pc*O6 colonization (Fig 4). With BODIPY, the images of control and *Pc*O6—colonized roots (Fig 5) show green fluorescence, indicative of lipid peroxides, for cells primarily in the distal root cap region. The root tips grown with CuO NPs showed epidermal damage and increased green fluorescence in cells in the root cap and in the root hairs (Fig 5). Root tips from *Pc*O6—colonized and CuO NP-exposed seedlings were stained similarly (Fig 6). With DHE, which detected superoxide anions, discrete cells in the 1.5 mm root tip segments displayed red fluorescence for each of the treatments especially in the root cap (Fig 6). The overall intensity of red fluorescence was greater for cells in the roots grown with CuO NPs, with or without *Pc*O6 root colonization. The root hairs for plants grown with CuO NPs showed fluorescence only in a discrete zone presumably at the site of the nucleus (Fig 6). The results from the use of DHE were similar to those with CellROX Deep Red, which according to the manufacturer detects superoxide anions but also intracellular hydroxyl radicals.

Damage to the epidermis, visible as a curling back of the root cap layer, was observed in some of the CuO NPs-treated samples as shown in the image for the CuO NPs-*Pc*O6 treatment (Fig 5). Similar damage to the root epidermis was reported in cowpea from Cu ions [41]. Also when *Pc*O6 was present in seedlings grown with CuO NPs the distance between the root tip and the zone of elongation was visibly greater (Figs 4–6).

**Fig 7:**
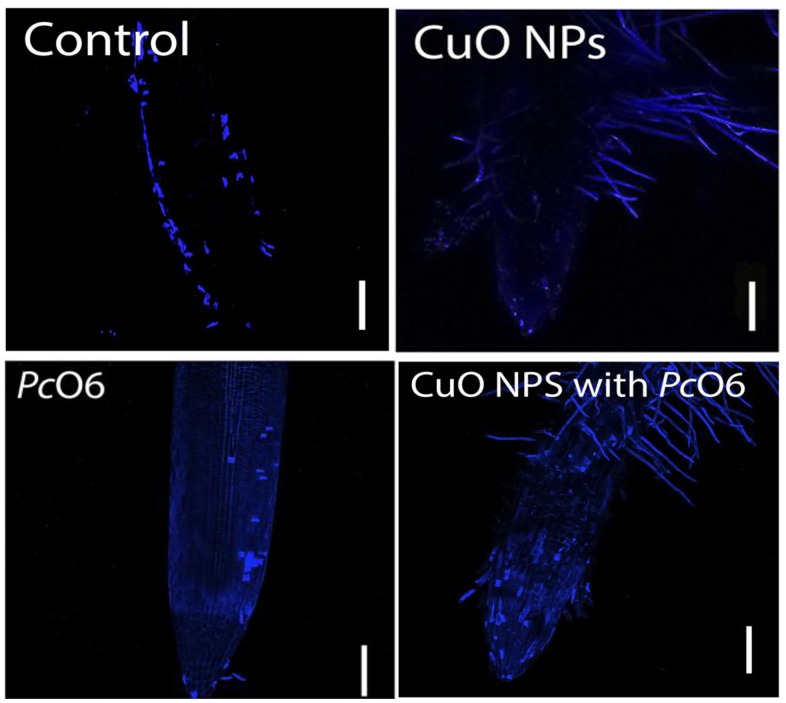
Staining for dead plant cells using SYTOX blue. For each study at least three root tips were examined for one treatment and at least three independent studies were performed before representative images were selected. The bar represents 200 μm.

Dead cells at the surface of the root tip were imaged as fluorescent blue cells using the fluorescent dye, SYTOX Blue. There was no overall protective effect noted for roots colonized by *Pc*O6 with or without CuO NPs exposure (Fig 7).

### Effects of *Pc*O6 on the composition of organic acids in the rhizosphere solution

**Fig 8:**
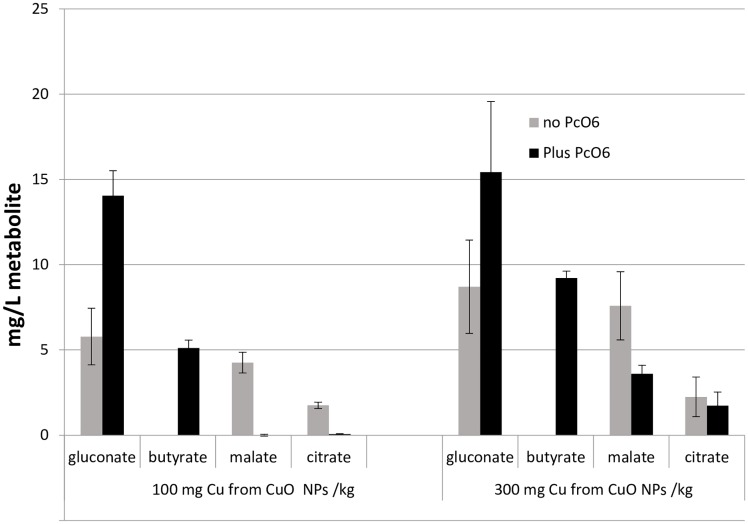
Changes in organic acids detected in the rhizosphere solutions from wheat seedlings grown for 7 d with addition to the sand of CuO NPs at two doses (100 and 300 mg/kg sand) for roots with and without *Pc*O6 colonization. The data are means and standard deviations from two studies each with 5 replicates of growth boxes containing 20 plants. The accumulations of the organic acids are statistically different at p = 0.05 for treatments with *Pc*O6 compared to those without.

Colonization of roots by *Pc*O6 in wheat seedlings grown with CuO NPs resulted in higher levels of gluconate and the presence of butyrate in the rhizosphere solution compared to the metabolites from plants exposed to CuO NPs without colonization. In studies where roots were grown in the absence of CuO NPs, the presence of *Pc*O6 cells increased gluconate content from 5.0 ± 0.5 mg/L to 11.0 ± 0.5 mg/L; no butyrate was detected in the absence of *Pc*O6. In contrast, the levels of two strong Cu chelators, malate and citrate, decreased in the rhizosphere solution when the roots grown with CuO NPs were colonized by *Pc*O6 (Fig 8). Other low molecular weight organic acids (formate, oxalate and acetate) were detected but with no consistent change due to treatments (data not shown).

### Influence of PcO6 colonization on expression of genes in root cells

Q-RT-PCR was used to assess how accumulation in the wheat roots of RNA from stress-related genes varied in the presence and absence of CuO NPs and root colonization with *Pc*O6. The highest increases in gene expression were observed in RNA extracted from wheat roots grown with CuO NPs (Table 1). Reduced accumulations of RNA transcribed for these genes was observed in extracts from roots of *Pc*O6 -colonized roots exposed to CuO NPs compared to the levels in roots grown with CuO NPs. The exception was for a gene for lipoxygenase, where transcript levels were not modified under any of the treatments, and a gene for defensin, where induction by CuO NPs was not altered by *Pc*O6 colonization. Colonization by *Pc*O6 alone did not induce the genes studied.

**Table 1:** Changes in gene expression (log_2_) in root tissues from 7 d-old wheat seedlings grown in 300 mg/kg sand Cu from CuO NPs with and without root colonization by *Pc*O6 compared with roots grown with *Pc*O6 and no Cu challenge. All data were normalized using expression from the gene encoding the wheat ADP ribosylation factor. Data are means from two independent growth studies where RNA from three roots for each treatment was extracted for Q-PCR analysis.

Gene function	CuO NPs	*Pc*O6	CuO NPs plus *Pc*O6
**Metal stress- associated**			
Metallothionein	12.5 ± 0.5	0.9 ± 0.1	6 ± 1
Chemocyanin	24 ± 9	1.0 ± 0.1	9 ± 2
Blue copper binding protein	23 ± 3	0.9 ± 0.1	7 ± 2
**Defense**			
Protease-inhibitor protein	10 ± 2	0.9 ± 0.4	4.2 ± 1
Defensin	13 ± 1	1.1 ± 0.1	12 ± 2
Xylanase inhibitor- like	4.2 ± 0.2	0.8 ± 0.1	2.3 ± 0.1
Beta—glucanase	21 ± 7	1.0 ± 0.1	8.5 ± 5
**ROS stress related**			
Lipoxygenase	1.3 ± 0.5	0.5 ± 0.4	1.1 ± 0.1
Glutathione transferase	13 ± 3	1.3 ± 0.4	4.5 ± 0.5
**ABA regulated**			
Cold acclimation proteinWCOR80	24 ± 4	0.9 ± 0.1	9.1 ± 1.0
ABA-associated protein	17 ± 10	0.8 ± 0.1	4.0 ± 0.5
Phosphoethanolamine Methytransferase	5 ± 2	1.0 ± 0.1	3 ± 1
**Regulatory factors**			
WRKY factor	20.4	0.7	12.7
Zinc finger protein	17.5	0.6	10.9

High increases in accumulation were observed for genes encoding structures related to three metal-binding proteins, metallothionein, chemocyanin and a blue copper-binding protein. A gene encoding glutathione transferase involved in glutathione homeostasis that relates to metal and oxidative stress was upregulated similarly. There also was upregulation of genes encoding proteins involved in pathogen defense, inhibitors for protease and invertase and ß-glucanase activities, and in water-stress related proteins.

## Discussion

The extent of protection conferred by root colonization with *Pc*O6 from the CuO NPs-induced toxicity in wheat seedlings was minimal when only morphology was examined but was considerable for gene expression and demonstrable for changes in the composition of rhizosphere metabolites. There was a trend for the root tip to root hair distance to be increased for the plants grown with CuO NPs when colonized by *Pc*O6. Collectively these data showed that the challenge to the plant imposed by CuO NPs remained dominant but that it was modulated by the presence of the root-colonizing bacterial cells.

Based on the assessment of culturable colonies released from the roots, there was no effect of the CuO NPs on *Pc*O6 colonization. Similar findings have been previously reported: for *Pc*O6 and bean challenged with CuO NPs [38] and in another study where peroxyacetic acid was used as an oxidative stress challenge to colonization by an isolate of *P*. *putida* and its mutant lacking a major catalase [42]. Protection may be due to the bacterial cells being embedded in a protective matrix such as that observed in SEM imaging of the colonized wheat roots. Resilience of biofilm cells to stress is a common phenomenon [43]. Studies with another beneficial microbe suggest that interaction between the bacterial cells and the NPs could enhance bacterial cell attachment to a root surface [44]. This observation was for colonization of oil seed rape root surfaces by a *Bacillus amyloliquefaciens* isolate grown in an agar matrix in the presence of titania NPs [44]. Unlike the interaction of CuO NPs with *Pc*O6 in planktonic suspensions, the titania NPs did not reduce growth of the *Bacillus* isolate [44].

The use of fluorescent dyes confirmed other reports that exposure to CuO NPs increased ROS stress in challenged plant cells [12, 16, 23, 45]. A novel finding was the detection of enhanced accumulations of superoxide anions, which could account for the intracellular ROS and the lipid peroxides especially in the root cap cells and root hairs. The findings that CuO NP-induced ROS were little altered by *Pc*O6 colonization suggested that the *Pc*O6 ROS-degrading enzymes had no impact on ROS stress in the root tip and that the ROS generated did not affect *Pc*O6 cell survival. This fact was supported by the ability of the *gacS* mutant, with impaired levels of catalase and superoxide dismutase [24, 25], to colonize the roots with and without the added stress of CuO NPs in the growth medium. The slight increase in intracellular ROS for the root tips grown with *Pc*O6 colonization could be due to the recognition, by an innate immunity response to MAMP-like materials, such as lipopolysaccharide or flagellin produced by the bacterium [46]. The slightly elevated levels of ROS also could be due to production by *Pc*O6 of the volatile fermentation product, 2R,3R- butanediol, which rapidly induced both hydrogen peroxide and nitric oxide in plant tissues [46, 47].

Stress in the root cells for plants grown with CuO NPs was displayed by activation of a wide array of defense-associated genes. The proposed function of the gene products illustrated overlap in protection against different stresses. For example, glutathione-S-transferase is proposed to target organic hydroperoxides [48], and increased expression of members of the gene family has been observed with metal and pathogen challenges to plant tissues [49]. Roles in resistance to pathogen attack have been designated for genes with strongly increased expression encoding proteins such as the plant defensins [50], beta-glucanases [51] and xylan-inhibitor proteins [52]. Protective responses against water stress and salinity are documented for ABA-regulated proteins, cold acclimation protein such as WCOR80 and phosphoethanolamine methyltransferase (PEMT) [53]. Among the genes most strongly induced were those anticipated to function with protective roles against metal challenges. These genes encoding proteins associated with metal-binding, i.e., metallothionein, chemocyanin and blue copper binding protein. The exact function of these proteins has not been resolved: metallothineins have been proposed to aid metal storage and chaperone metals into apoproteins whereas the chemocyanins may function as chelators for transport of metals into vacuoles as a protective measure [54]. In wheat, expression of a chemocyanin-like gene was controlled by a small microRNA; increased expression with Cu ion stress, salt exposure and resistance to pathogens correlated with decreased expression from the microRNA gene [55]. A transcriptome study in *Arabidopsis* showed that a blue copper protein gene was among those most highly induced in roots challenged with CuO NPs [45]. These examples illustrate the complexity of cell signaling pathways that could be involved in the plants responses to Cu. Indeed, a gene encoding one of the beta-glucanase family of proteins in wheat was enhanced by three cell regulators, salicylic acid, methyl jasmonate and ethylene [51].

The reduction in expression of the CuO NP-induced genes conferred by *Pc*O6 root colonization generally was within a two- to three- fold range, suggesting that a global mechanism accounted for the change in level. The finding that none of the genes examined had expression stimulated by *Pc*O6 colonization alone, was consistent with the concept of priming, where the beneficial microbe alone does not induce the gene but rather it primes responses when the plant is stressed [56]. Reduced gene expression observed with *Pc*O6 colonization and the CuO NP challenge could be due to a combination of factors. There could be physical exclusion caused by the matrix of material observed to be surrounding bacterial cells associated with the root surface. Bacterial surface features and metabolites could alter defense signaling in the plant. For instance, *Pc*O6 induces the jasmonate/ethylene pathways for defense stimulation while reducing defense pathways associated with salicylic acid [57]. The protected plants also may be demonstrating growth stimulation by the IAA generated by *Pc*O6 from plant-supplied tryptophan. IAA-induced responses are one of the mechanisms proposed for improved plant growth on metal-contaminated soils [5]. *Pc*O6 has the potential to produce IAA from tryptophan in culture [19] and on root surfaces [58]. Future work should explore another possibility, a role for the siderophores produced by the bacterium. *Pc*O6 produces a pyoverdin-like siderophore that will chelate other metals as well as iron [20]. Bacterial siderophore production is cited in phytoremediation studies as being an additional trait of significance for plant metal tolerance [5]. However, lower solubility of Cu was observed in the rhizosphere solution for *Pc*O6 –colonized plants and this reduced level may have modified the gene expression levels in the roots.

The lower soluble Cu detected in the rhizosphere could be due to physical sorption of the NPs and any Cu released from dissolution of the NPs onto the matrix observed around attached bacterial cells or to the bacterial cell surfaces. Sorbance of Cu ions to cell surfaces of another pseudomonad was observed [59]. Thus, the bacteria would act as a physical shield limiting the impact of Cu on the plant root. Secondly, reduction in the level of two major metal chelators [31], citrate and malate, in the rhizosphere solution may be important. We postulate that these organic acid were consumed as nutrients by *Pc*O6, as suggested by growth of the cells in culture using the acids as sole carbon sources (data not shown). The lower levels of these organic acids could have contributed directly to reduced dissolution of Cu from CuO NPs and led to modified levels of Cu uptake into the wheat shoots. The release of Cu from CuO NPs has been shown to be greatly enhanced by citrate [60].

We speculate that the increase in butyrate in the rhizosphere solutions of the *Pc*O6-colonized plants could be due to fermentation of acetyl CoA as predicted in MetaCyc based on genome annotation; *Pc*O6 has another demonstrated fermentative pathway, the production of 2, 3, butandiol [45]. The origin of gluconate is uncertain because no pathways for gluconate production have been characterized for plant metabolism. However, gluconate is a predicted product from an alternative pathway for glucose oxidation being formed spontaneously from D- glucono- 1,5- lactone generated by a ubiquinone-dependent glucose dehydrogenase. Such enzymes have been detected in bacteria and fungi. The genome of *Pc*O6, has genes that would encode proteins with these functions at the loci PchlO6_1551 and PchlO6_4960. *Pc*O6 also has additional genes encoding enzymes for the degradation of gluconate to gluconate- 6- phosphate and further catabolism of this metabolite by the Entner-Douderoff pathway. Understanding of the contribution of *Pc*O6 to gluconate levels in the rhizosphere awaits genetic analysis using specific *Pc*O6 mutants blocked in gluconate synthesis and degradation.

In conclusion, microbes associated with root surfaces modify plant responses to CuO NPs. In soil where a root will have a mixture of microbes at the root surface, these interactions will become more complex than in the gnotobiotic model sand system used in this paper. Protection against the toxicity of the CuO NPs also was manifest for the bacteria as they colonized the root surface. The interactions that achieve protection likely are multifaceted. The mechanisms may include physical shielding of plant root surfaces by the bacteria, the catabolism of metabolites in plant root exudates involved in dissolution of the CuO NPs, the production of bacterial products that alter plant function and bioavailability of Cu from CuO NPs, and the production of cell signaling metabolites that regulate both bacterial and plant metabolism.

## Supporting Information

S1 Fig Effect of *Pc*O6 root colonization on shoot height in the presence of 300 mg Cu from CuO NPs/kg sand. (DOCX)

S2 Fig Culturability of cells of wild type *Pc*O6 or its *gacS* mutant recovered from 7 d-old wheat seedlings grown in the presence of 0, 100 or 300 mg Cu from CuO NPs. (DOCX)

S3 Fig Association of Cu with unwashed coleoptiles after growth in sand with CuO NPs. /kg sand. (DOCX)

S4 Fig Contamination of outer but not inner surfaces of coleoptiles with CuO NPs. (DOCX)

S5 Fig Effect of CuO NPs addition to growth matrix on coleoptile growth. (DOCX)

S6 Fig Background fluorescence of wheat root tips under imaging conditions used for fluorescence from DHE and Sytox Blue (SB), BODIPY (BD) and Dark Red (DB). (DOCX)

S1 Table Gene specific primer pairs used for qRT-PCR. (DOCX)
