# Supplementary material for: A Root-Colonizing Pseudomonad Lessens Stress Responses in Wheat Imposed by CuO Nanoparticles
Source: PLoS One. 2016 Oct 24;11(10):e0164635. doi: 10.1371/journal.pone.0164635 (PMC5077138; doi:10.1371/journal.pone.0164635)

**S1 Fig. Effect of *Pc*O6 root colonization on shoot height in the presence of 300 mg Cu from CuO NPs/kg sand.** Data are from one study with 5 replicates each with 5 wheat seedlings /growth box and are typical of at least two other studies


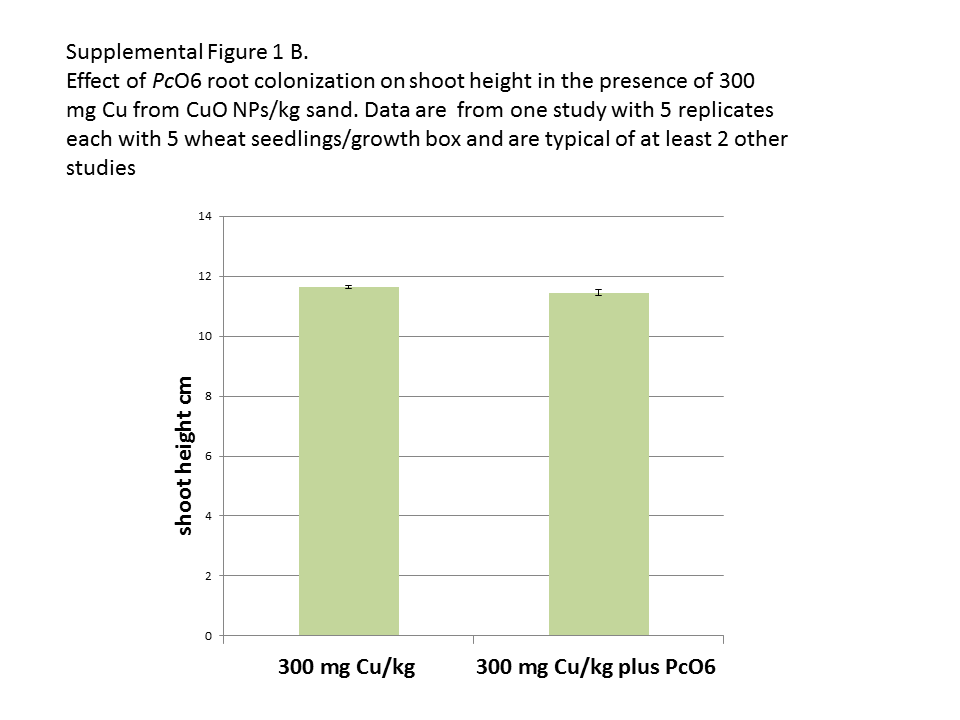

Supplement: S1 Fig — (DOCX) [file pone.0164635.s001.docx]
