# Supplementary material for: A Root-Colonizing Pseudomonad Lessens Stress Responses in Wheat Imposed by CuO Nanoparticles
Source: PLoS One. 2016 Oct 24;11(10):e0164635. doi: 10.1371/journal.pone.0164635 (PMC5077138; doi:10.1371/journal.pone.0164635)

**S2 Fig. Culturability of cells of wild type *Pc*O6 or its *gacS* mutant recovered from 7 d-old wheat seedlings grown in the presence of 0, 100 or 300 mg Cu from CuO NPs/kg sand.** Data are means with standard errors of three roots harvested from three separate growth boxes.


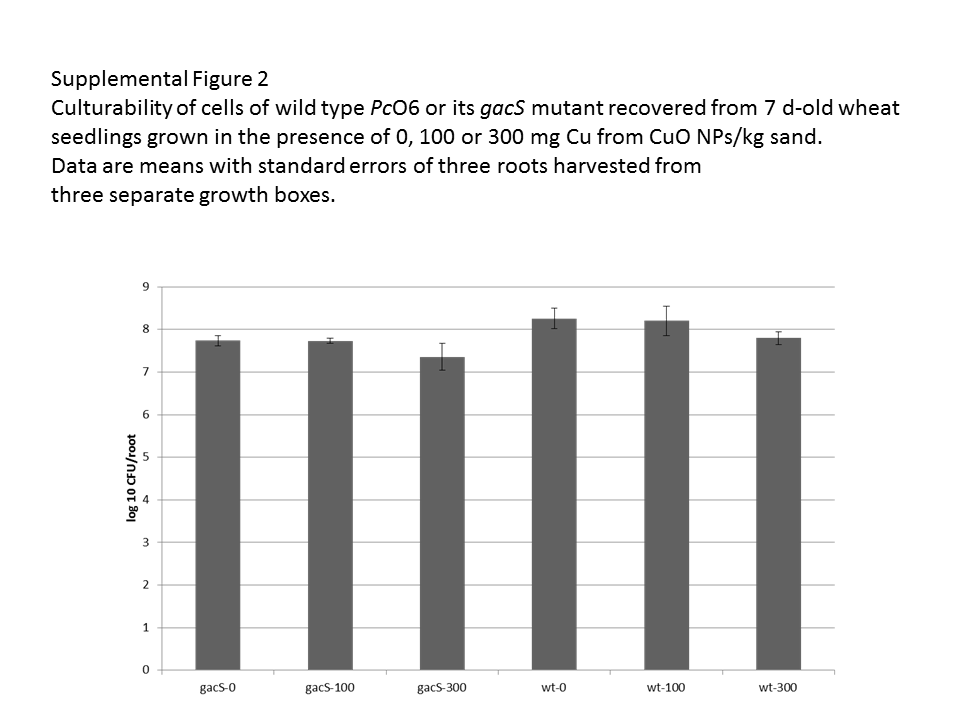

Supplement: S2 Fig — (DOCX) [file pone.0164635.s002.docx]
