# Supplementary material for: A Root-Colonizing Pseudomonad Lessens Stress Responses in Wheat Imposed by CuO Nanoparticles
Source: PLoS One. 2016 Oct 24;11(10):e0164635. doi: 10.1371/journal.pone.0164635 (PMC5077138; doi:10.1371/journal.pone.0164635)

**S3 Fig. Association of Cu with unwashed coleoptiles after growth in sand with CuO NPs.** The data are means and standard deviations obtained from seedlings grown for 7 d in 5 replicated growth box each with 5 plants.


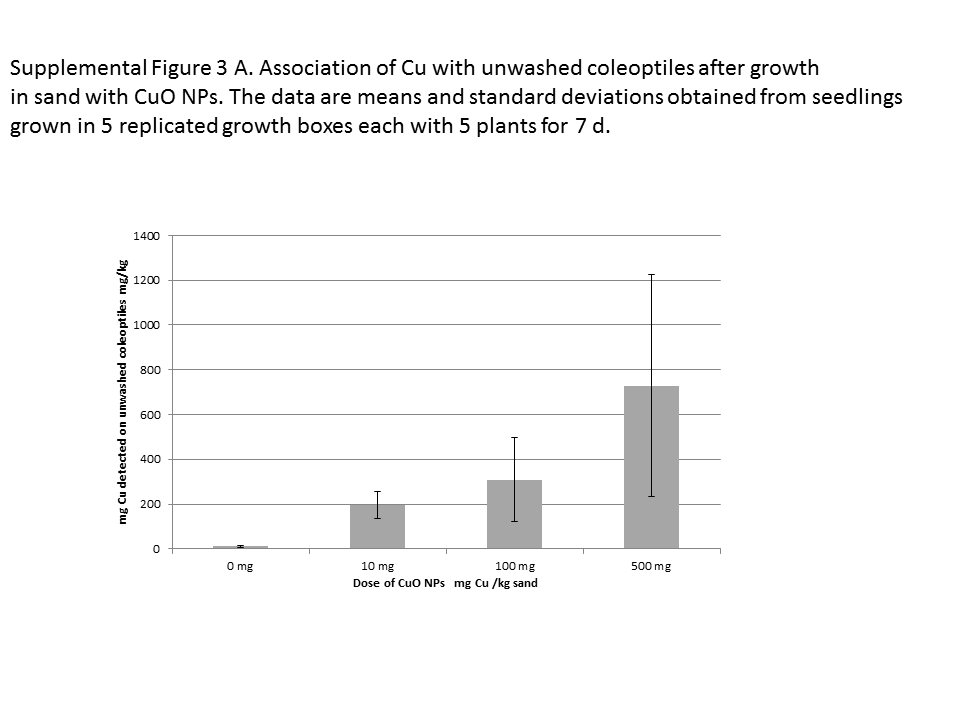

Supplement: S3 Fig — (DOCX) [file pone.0164635.s003.docx]
