# Supplementary material for: A Root-Colonizing Pseudomonad Lessens Stress Responses in Wheat Imposed by CuO Nanoparticles
Source: PLoS One. 2016 Oct 24;11(10):e0164635. doi: 10.1371/journal.pone.0164635 (PMC5077138; doi:10.1371/journal.pone.0164635)

**S4 Fig. Contamination of outer but not inner surfaces of coleoptiles with CuO NPs.** SEM images are shown for the inner and outer surfaces. The EDS analysis of one particle is typical of six other particles examined from three different coleoptiles.


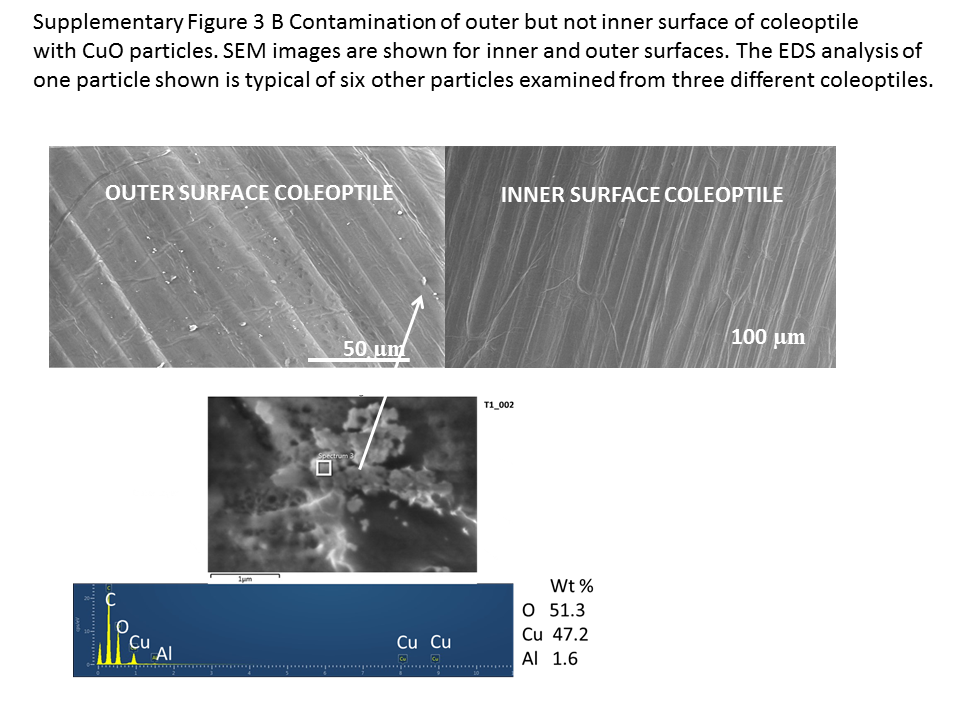

Supplement: S4 Fig — (DOCX) [file pone.0164635.s004.docx]
