# Supplementary material for: A Root-Colonizing Pseudomonad Lessens Stress Responses in Wheat Imposed by CuO Nanoparticles
Source: PLoS One. 2016 Oct 24;11(10):e0164635. doi: 10.1371/journal.pone.0164635 (PMC5077138; doi:10.1371/journal.pone.0164635)

**S5 Fig. Effect of CuO NPs addition to growth matrix on coleoptile growth.** The data shown are from one of three studies with similar results. The mean of the lengths of the coleoptiles with standard deviations are shown for plants harvested from 5 replicated growth boxes each with 5 plants/box after 7 d growth.


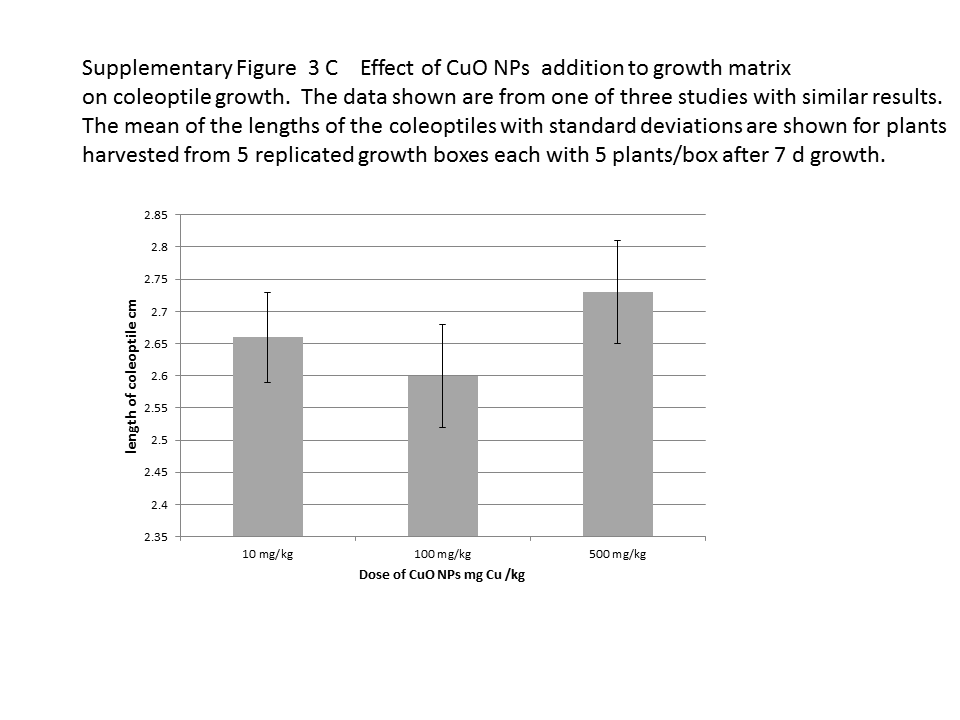

Supplement: S5 Fig — (DOCX) [file pone.0164635.s005.docx]
