# Supplementary material for: A Root-Colonizing Pseudomonad Lessens Stress Responses in Wheat Imposed by CuO Nanoparticles
Source: PLoS One. 2016 Oct 24;11(10):e0164635. doi: 10.1371/journal.pone.0164635 (PMC5077138; doi:10.1371/journal.pone.0164635)

**S6 Fig. Background fluorescence of wheat root tips under imaging conditions used for fluorescence from DHE and Sytox Blue (SB), BODIPY (BD) and Dark Red (DB).** Treatments are as described in Materials and Methods


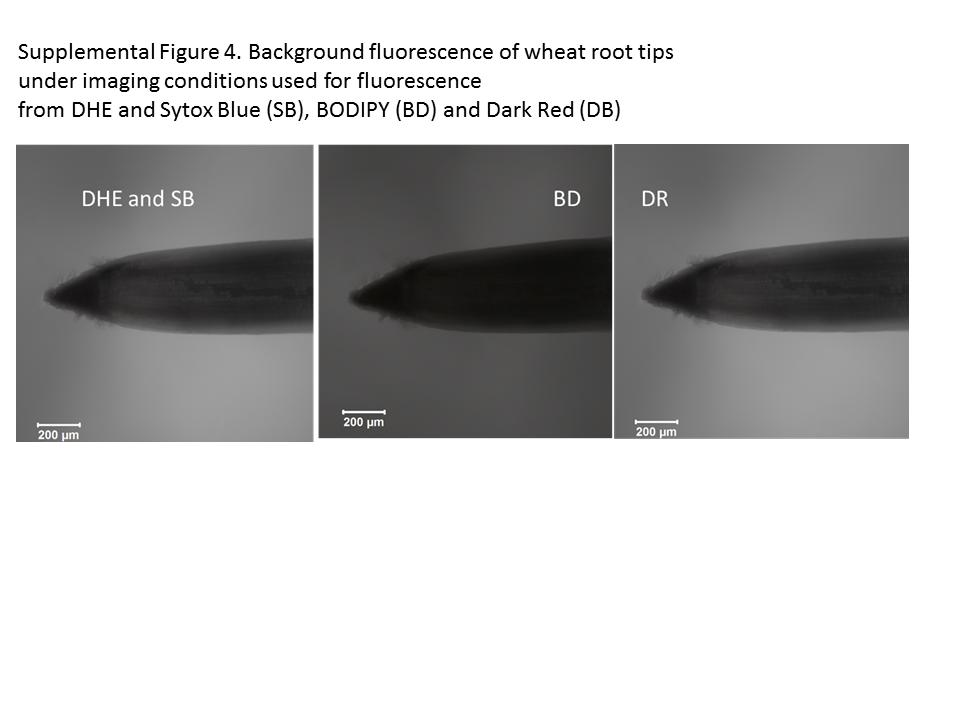

Supplement: S6 Fig — (DOCX) [file pone.0164635.s006.docx]
