# Supplementary material for: A Root-Colonizing Pseudomonad Lessens Stress Responses in Wheat Imposed by CuO Nanoparticles
Source: PLoS One. 2016 Oct 24;11(10):e0164635. doi: 10.1371/journal.pone.0164635 (PMC5077138; doi:10.1371/journal.pone.0164635)
Supplement: S1 Table — (DOCX) [file pone.0164635.s007.docx]

**S1 Table** **Gene specific primer pairs used for qRT-PCR**

| **Potential function, gene name and identification** | **Forward primer (5′-3′)** | **Reverse primer (5′-3′)** |
| --- | --- | --- |
| Wheat ADP-ribosylation factor | ccgtgttgttgaagcaaggg | tctcagcagcattcatggca |
| **Metal stress- associated** |  |  |
| Metallothionein  A_99_P22551  GenBank AY736124 | gtaccctgatctgacggagc | cacttgcagttgtcgccg |
| Chemocyanin  A_99_P200731  GenBank AK330966 | aaataaaatgcccgcgtccc | actgcacacaaaacacgctc |
| Blue copper-binding protein  A_99_P226086  GenBank FJ459810,  DR 740012 | cgccatgaagatcaccctcc | acacccagttgctgtagtcg |
| **Defence** |  |  |
| Proteinase inhibitor protein  A_99_P234306  GenBank AY549888 | acaactgcaggtccttctcc | ccatcgacggtgctcttcat |
| Defensin  A_99_P254206  GenBank BJ293335 | cctcatcgtgctcctcctc | gcagttggtgtcgctgaag |
| Wheat_ Xylanase inhibitor-like  A_99_P268711  GenBank AK332666s | tggagaacaagctggtggtg | cggccctggtgaaattgaag |
| Beta – glucanase  A_99_P17814  GenBank DQ090946 | aacgtgtacccctacttcgc | gcgtcgaacagggatgtgta |
| **ROS stress-related** |  |  |
| Lipoxygenase  A_99_P234306  GenBank AK 332064.1 | atcctgtccaagcactcctc | ccaccacctgcttctcgatg |
| Glutathione transferase  A_99_P423722  GenBank JX051004 | ttcaagacgtgctccaaggg | gtcgtcgaaaagccgcatg |
| **ABA regulated** |  |  |
| Cold acclimation proteinWCOR80  A_99_P167849 | ccggcgagaagaaaggca | tggtgtcatgcatctccgtg |
| ABA-inducible protein WRAB1  A_99_P2118621  AF139915.1 | cagtacaccaaggagtccgc | tggcattgtcaccacccatg |
| Phosphoethanolamine methyltransferase  A_99_P223461  GenBank AK332332.1 | atgcttgaggatgccggttt | tgttcttctctgtctcgccc |
| **Regulatory factor** |  |  |
| WRKY 19 a  A_99_{148637  GenBank AK335019.1 | ggattcagtggccggtagag | catactccatgtcagccccg |
| C2 H2 Zinc finger  A_99_P177186  GenBank EU408224.1 | caagaggagaagcagcacga | ccacacccactgatcgcc |
